# A prospective study of HER3 expression pre and post neoadjuvant therapy of different breast cancer subtypes: implications for HER3 imaging therapy guidance

**DOI:** 10.1186/s13058-024-01859-w

**Published:** 2024-06-29

**Authors:** Nicoleta Sinevici, Christine E. Edmonds, Brian N. Dontchos, Gary Wang, Constance D. Lehman, Steven Isakoff, Umar Mahmood

**Affiliations:** 1 Department of Radiology, Massachusetts General Hospital and Harvard Medical School, 149 13th Street, Boston, MA USA; 2 Department of Hematology and Oncology, Massachusetts General Hospital and Harvard Medical School, Boston, MA USA

**Keywords:** HER3, Neoadjuvant, Breast cancer, Therapy, Imaging, Resistance, Immune suppressive

## Abstract

**Purpose:**

HER3, a member of the EGFR receptor family, plays a central role in driving oncogenic cell proliferation in breast cancer. Novel HER3 therapeutics are showing promising results while recently developed HER3 PET imaging modalities aid in predicting and assessing early treatment response. However, baseline HER3 expression, as well as changes in expression while on neoadjuvant therapy, have not been well-characterized. We conducted a prospective clinical study, pre- and post-neoadjuvant/systemic therapy, in patients with newly diagnosed breast cancer to determine HER3 expression, and to identify possible resistance mechanisms maintained through the HER3 receptor.

**Experimental design:**

The study was conducted between May 25, 2018 and October 12, 2019. Thirty-four patients with newly diagnosed breast cancer of any subtype (ER ± , PR ± , HER2 ±) were enrolled in the study. Two core biopsy specimens were obtained from each patient at the time of diagnosis. Four patients underwent a second research biopsy following initiation of neoadjuvant/systemic therapy or systemic therapy which we define as neoadjuvant therapy. Molecular characterization of HER3 and downstream signaling nodes of the PI3K/AKT and MAPK pathways pre- and post-initiation of therapy was performed. Transcriptional validation of finings was performed in an external dataset (GSE122630).

**Results:**

Variable baseline HER3 expression was found in newly diagnosed breast cancer and correlated positively with pAKT across subtypes (r = 0.45). In patients receiving neoadjuvant/systemic therapy, changes in HER3 expression were variable. In a hormone receptor-positive (ER +/PR +/HER2-) patient, there was a statistically significant increase in HER3 expression post neoadjuvant therapy, while there was no significant change in HER3 expression in a ER +/PR +/HER2+ patient. However, both of these patients showed increased downstream signaling in the PI3K/AKT pathway. One subject with ER +/PR −/HER2− breast cancer and another subject with ER +/PR +/HER2 + breast cancer showed decreased HER3 expression. Transcriptomic findings, revealed an immune suppressive environment in patients with decreased HER3 expression post therapy.

**Conclusion:**

This study demonstrates variable HER3 expression across breast cancer subtypes. HER3 expression can be assessed early, post-neoadjuvant therapy, providing valuable insight into cancer biology and potentially serving as a prognostic biomarker. Clinical translation of neoadjuvant therapy assessment can be achieved using HER3 PET imaging, offering real-time information on tumor biology and guiding personalized treatment for breast cancer patients.

**Supplementary Information:**

The online version contains supplementary material available at 10.1186/s13058-024-01859-w.

## Introduction

Breast cancer remains a leading cause of cancer related death worldwide, with a lifetime incidence rate of one in eight women [1]. HER3 is a member of the epidermal growth factor receptor (EGFR) family (EGFR, HER2, HER3 and HER4) and has been implicated in tumor initiation, progression and resistance, a major contributor to treatment failure [2, 3]. Prior research has demonstrated associations between HER3 expression and metastasis, histological grade, tumor size, and recurrence [4, 5].

HER3 is a pseudokinase relying on its family members to induce phosphorylation and cell signaling through the downstream PI3K/AKT and RAS/RAF/MAPK pathways [6]. HER3 is endowed with 6 binding sites for the p85 subunit of the PI3K, making it a potent activator of this pathway [7]. Compared to the other family members HER3 can dimerize with other non-HER receptors such as mesenchymal-epithelial transition factor (MET) receptor and fibroblast growth factor receptor 2 (FGFR2). Furthermore, HER3 can activate other signaling pathways including janus kinase (JAK) and proto-oncogene c-Src (SRC) involved in cancer proliferation [8]. Multiple regulation mechanisms of HER3 signaling have been described including transcriptional, post-transcriptional, translational, post-translational, and localizational control [7].

HER3 activation enhances the metastatic potential of cancer cells, causing treatment resistance [9–12]. In HER2 + breast cancer, drug exposure can lead to HER3 mediated compensatory signaling, leading to a 2-log fold increase in signaling output, far exceeding the therapeutic index of drugs acting on the HER2 active kinase domain [13]. HER3 has been implicated in breast cancer resistance to multiple therapies, including antiestrogen therapies fulvestrant and tamoxifen, EGFR and HER2 small molecule inhibitors such as erlotinib and lapatinib, and HER2 directed antibodies such as Herceptin (trastuzumab) and Pertuzumab [4, 14, 15]. In patients with triple negative breast cancer (TNBC), combined high HER3 and EGFR expression was associated with worse 10- year survival after chemotherapy [16].

In the era of personalized medicine, identification of patients in which HER3 should be therapeutically targeted has led to the development of several methods for HER3 interrogation. Due to the dynamic nature of HER3 expression as well as the current limitations of biopsy assessment (such as tumor heterogeneity, repeat sampling, temporal changes), a promising avenue is the molecular imaging of HER3 via Positron Emission Tomography (PET). PET imaging offers a number of advantages including real-time, non-invasive repeat imaging. Our group and others have demonstrated the clinical potential of HER3-PET imaging in respect to feasibility and accuracy in preclinical [17] and clinical studies [18, 19]. The current field of HER3 imaging has been reviewed in [20].

In appropriately selected patients, HER3 targeted therapies hold promise in combination therapy regimens, particularly in the neoadjuvant setting [12]. Anti-HER3 therapeutics are currently under investigation in Phase 1/2 clinical trials for treatment of different breast cancer subtypes, including HR + /HER2- [21], HER2+ [22] and TNBC (NCT05057013).

In this prospective clinical study, we investigate the baseline expression of HER3 in newly diagnosed breast cancer, as well as the expression of other EGF family proteins and proteins in the PI3K pathway, by breast cancer subtype. Patients with ER + , PR ± , HER2- were classed as ER + ; cancers that showed HER2 + expression were classed as HER2 irrespective of their hormonal status; and cancers that showed no expression of these three receptors were classed as TNBC. In addition, in a small subset of these cases, we conduct a preliminary exploration of the effect of neoadjuvant therapy (NAT) on HER3 expression. We further explore the role of HER3 after NAT in a publicly available transcriptomic dataset (n = 34). Interestingly, we report for the first time an immunosuppressive microenvironment in patients with increased HER3 expression post NAT.

Detection of HER3 and downstream signaling proteins following initiation of NAT may implicate feedback mechanisms that occur early in the therapeutic response, which may significantly alter treatment response and disease progression.

## Materials and methods

### Patient selection

The study was conducted between May 25, 2018 and October 12, 2019. The study was performed with institutional review board approval, and informed consent was obtained from all enrolled patients. The study complies with the Health Insurance Portability and Accountability Act.

Non-pregnant patients ages 18 and above were eligible for the study if they had a breast mass on any breast imaging modality (mammography, sonography, or breast MRI) measuring at least 2 cm in greatest dimension, that was deemed highly suspicious (greater than 90% chance) by a breast radiologist for invasive breast carcinoma. Beyond the requirement for a tumor of at least 2 cm in size, there were no other stage inclusion or exclusion criteria, so those enrolled include both locally advanced and stage IV/metastatic breast cancer.

Patients were excluded from the study if they had a previously treated breast cancer, or if they had any concurrent malignancy diagnosis. Patients were recruited and enrolled in the study prior to undergoing their routine ultrasound guided core biopsy for cancer diagnosis, by a breast radiology attending physician who was also a member of this study team. Those enrolled in the study consented to undergo a ‘baseline’ research biopsy for the sole purpose of this study, which consisted of two additional 12-14G core needle biopsy specimens taken immediately after the routine clinical specimens were obtained.

Thirty-six total patients were enrolled in the study and underwent the baseline biopsy. Two of the 36 (5%) patients were removed from the final analysis; one was found to have benign pathology on the clinical breast biopsy, and the other patient had a tumor biopsy specimen too small for adequate analysis. Biopsies represent ER+ , HER2+ , and TNBC subtypes of breast cancer. The determination of the breast cancer subtype and receptor status was made per the routine clinical specimen analysis by breast pathology.

This study did not impact the treatment decisions of the breast oncology team. However, per study protocol, a member of the study team followed up the treatment plan for all study subjects following their baseline research biopsy. For all subjects who initiated systemic therapy prior to any breast surgery (either neoadjuvant therapy or, in the case of those diagnosed with Stage IV disease, palliative therapy), a clinician on the research study team reached out to the patients to recruit them to undergo a second (or follow-up) ultrasound guided core needle biopsy, to be performed on or between days 5 and 23 of systemic (neoadjuvant or palliative) therapy. Four subjects agreed to and underwent the follow-up research biopsy, which also consisted of a repeat ultrasound guided 12-14G core needle breast biopsy (of the same breast mass as that biopsied during the first research biopsy), with two core specimens again obtained.

The research biopsy specimens, including the baseline and the follow-up research biopsy specimens, were used for further analysis specific to the purposes of this research study. All research biopsy specimens were immediately flash frozen in liquid Nitrogen and stored at −80 °C until the time of the research laboratory analyses.

### Western blot analysis

For immunoblotting, tumor samples were lysed in RIPA buffer (Invitrogen) supplemented with protease and phosphatase inhibitors (Roche). Protein concentration was determined using the Bicinchoninic acid method (ThermoFisher). Equal protein amounts were separated by electrophoresis through Tris-glycine 4% to 20% gradient gels (Bio-Rad) and proteins were transferred onto PVDF membranes. Primary antibody incubation was performed overnight at 4 °C using the following antibodies: Anti-ER (8644, Cell Signaling), Anti-PR (8757, Cell Signaling), Anti-EGFR (2256, Cell Signaling), Anti-pEGFR (2234, Cell Signaling), anti-HER2 (SC52349, Santa Cruz), anti-pHER2 (2247, Cell Signaling), anti-HER3 (SC81455, Santa Cruz), anti-pHER3 (4791, Cell Signaling), anti-AKT (2920, Cell Signaling) anti-pAKT (4060S, Cell Signaling), anti-Pi3K (5405, Cell Signaling), anti-p-Pi3K (4228, Cell Signaling), anti-ERK (4695, Cell Signaling), anti-pERK (4377, Cell Signaling) and anti-GAPDH (ab9485, Abcam). The following day, blots were incubated for one hour in secondary antibody followed by chemiluminescent detection. Antibody detection and quantification were conducted using the iBright ™ FL1000 (ThermoFisher) and the iBright analysis software.

### Fluorescent immunohistology

Frozen biopsy samples were submitted to the Specialized Histopathology Services of the MGH Pathology Core for processing, sectioning and embedding. For immunohistofluorescence (IHF) staining, cores were fixed and permeabilized. Blocking was carried out for one hour at room temperature in PBS containing 5% goat serum. Primary antibody incubation was performed overnight at 4 °C with the addition of rabbit anti-mouse HER3 antibody (12,780, Cell Signaling). The following day, stained slides were washed and incubated with AlexaFluor Plus 647 conjugated goat anti-rabbit IgG secondary antibody (A32733; ThermoFisher) at room temperature in darkness for one hour. A coverslip was applied to each slide using Vectashield mounting medium for fluorescence microscopy with DAPI (4′, 6-diamidino-2-phenylindole). Quantification was performed by getting the mean of six complete and non-overlapping regions of interest (ROI) and dividing red channel signal (HER3) by the blue channel signal (DAPI). All images were acquired using Biotek Cytation 5 Cell Imaging Multi-Mode Reader and analyzed through Biotek Gen5 software.

### Quantitative RT-PCR

mRNA was isolated using the Dynabeads® (Thermofisher, MA) according to the manufacturer's instructions. cDNA was synthesized using VILO IV MasterMix (Thermofisher) per the manufacturer's instructions. qPCR was performed on an QuantStudio 3 thermal cycler using TaqMan Advanced MasterMix (Thermo Scientific). HER3 (Hs00176538_m1) and RPL-30 (Hs00265497_m1) qPCR primers sequences were obtained from Thermofisher. HER3 target gene expression was normalized to RPL-30.

### Bioinformatic analysis

To analyze HER3 mRNA expression in breast cancer, we queried the online database www.cbioportal.org. A total of 16 studies and 8925 patients were included in the search. The relationship of HER3 expression and disease free, progression free and overall survival was investigated. Corresponding Kaplan Meier plots were extracted and results are displayed with *p*-values obtained using the log-rank test. Co-occurrence data was analyzed by querying the expression of HER3 along with ESR, PRG, HER2 and EGFR, and results are displayed with *p*-values. *P*-values < 0.05 were considered significant. Associated signalling pathway networks and gene alterations are also reported.

Transcriptomic analyses were performed on the publicly available NEO study in the NCBI GEO data repository under accession GSE122630. This study consists of breast cancer patients with sequentially biopsied tumours at baseline (pre-treatment) (T0), at two weeks on treatment (T2), mid-treatment (T3), and at surgical resection (T4). Samples were of mixed histological grade and HER2 status [23]. Data analysis performed, including PAM50 calls [24], microenvironmental cell population analysis [25], DESeq2- differential gene expression [26], gene set enrichment analysis (GSEA) and single-sample gene set enrichment analysis (ssGSEA) [27] were performed in R using packages available through CRAN (http://cran.r-project.org/) and Bioconductor (http://www.bioconductor.org/).

### Statistical analysis

Statistical analysis were carried out using Prism v 7.0d or the R package. P-values < 0.05 were considered significant, unless otherwise stated.

## Results

### HER3 expression and pathway association

**Fig. 1:**
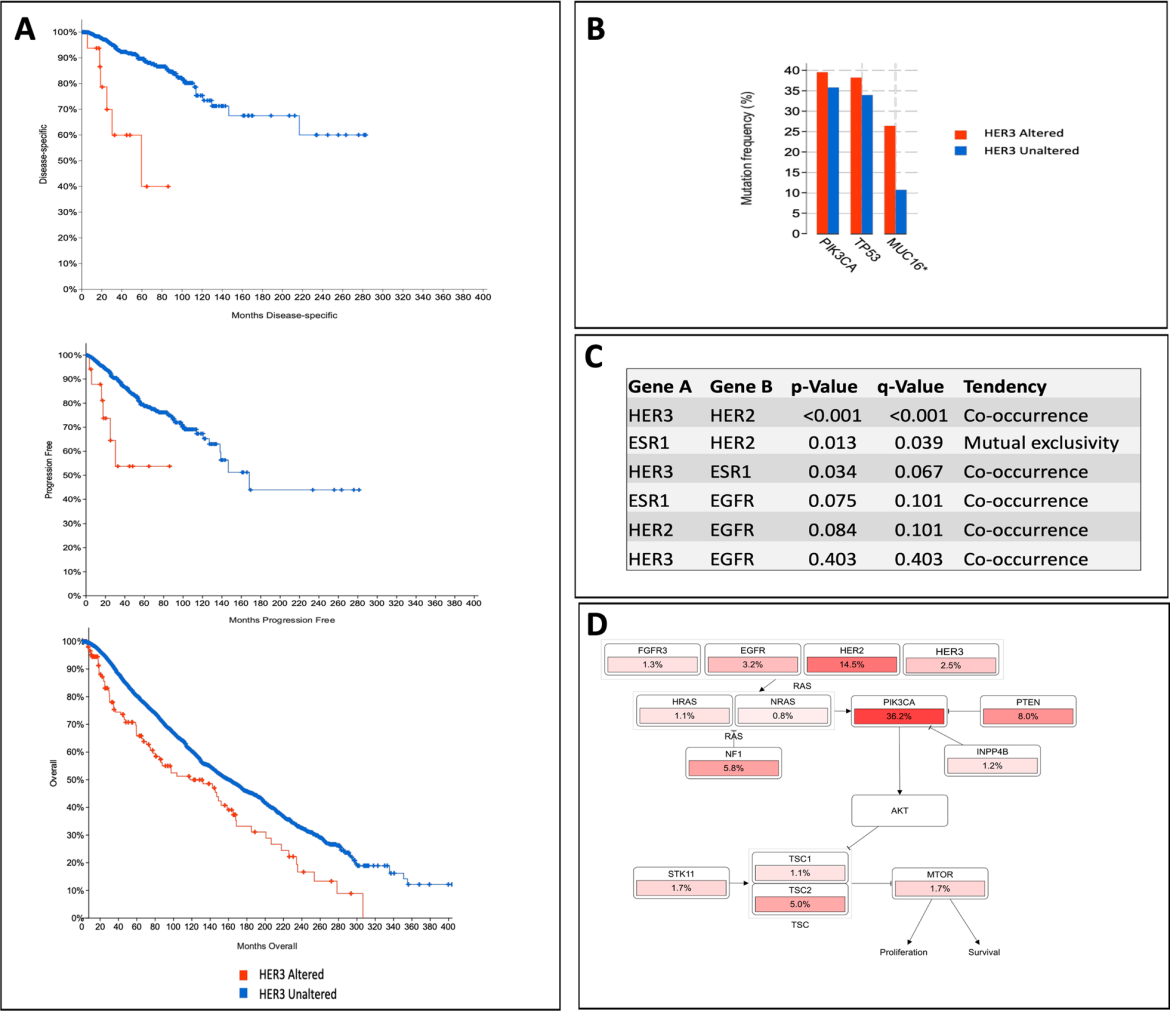
Survival analysis in the context of HER3 gene alteration. **A** Kaplan–Meier plots for disease-specific, progression free and overall survival of breast cancer showing HER3 gene alterations from 221 patients across 16 studies, using cBioPortal. **B** Mutation frequency in percentage of genes associated with HER3 gene signatures in breast cancer **C** alterations in EGFR, HER2, HER3 and ESR1 gene expression tendencies from patients with breast cancer **D** schematic representation of pathway signalling in patients with HER3 gene signature. Data was generated in (http://cbioportal.org)

Prior to laboratory analyses of the prospectively acquired patient biopsy specimens, we evaluated HER3 gene alteration (DNA mutations) in breast cancer patients, using the publicly available bioinformatic database, www.cbioportal.com. We found somatic mutation frequency of the HER3 gene to be low at 2% (n = 197), with most of the alterations being missense mutations (92%) and truncating mutations (5.5%), resulting in gain of function and amplification events in certain patients. We further analyzed the relationship between HER3 and patient outcome using Kaplan–Meier estimates up to 33-year follow-up in a series of 8926 total breast cancer patients, of whom 221 showed mutations in the HER3 receptor. Figure 1A shows that altered HER3 expression in breast cancer is associated with worse outcomes in terms of disease specific, progression free and overall survival. The median overall survival of patients with mutated HER3 breast cancer was 117.9 months compared to 160.4 in breast cancer patients with wild type HER3. The most significant altered gene mutations associated with HER3 were PI3K, p53, and MUC16 mutations (Fig. 1B). Mutations in these genes have been previously reported in numerous studies and are associated with worse prognosis in breast cancer [28–33].

We next queried the biological processes that may be associated with altered HER3 or other frequently expressed markers in breast cancer, including EGFR, HER2, ER and PR. Figure 1C shows that breast cancer alterations in these specific markers have a tendency for co-occurrence with HER3, meaning that genetic alteration in these genes are frequently found together with HER3 alterations in patients. The exception is between HER2 and ESR1 which show mutual exclusivity.

As expected, the PI3K/AKT/mTOR pathway as well as the RAS/RAF/MAPK pathway were associated with HER3 mutations in breast cancer (Fig. 1C). Dysregulation of individual nodes in the respective pathway at receptor level, e.g. EGFR/HER2, or in effector downstream signaling mediators, e.g. PI3K/AKT mutations, can contribute to continued signaling in the absence of HER3 activating mutations. No association was found between HER3 alterations and breast cancer subtype (receptor status) or clinical stage.

These observations demonstrate that altered HER3 expression is associated with aggressive forms of breast cancer and with poor clinical prognosis. Despite a relatively low frequency of genetic alterations, HER3 has been shown to be controlled at transcriptional, post-transcriptional, translational, post-translational and localized control [7], and thus normal HER3 levels may be sufficient to maintain cellular signalling [34].

## Patient characteristics

The clinicopathological characteristics of the patients enrolled in the study are shown in Table 1. A total of 34 patients were included in the analysis. 11.76% (4/34) patients had a diagnosis of invasive lobular carcinoma, 2.94% (1/34) had a mixed invasive lobular carcinoma and invasive ductal carcinoma, and 85% of patients were diagnosed with invasive ductal carcinoma with or without ductal carcinoma in situ. Of the total patients enrolled, 59.8% (20/34) were ER+, 32.4% (11/34) were classified as HER2+, and 8.8% (3/34) were classified as ER-/PR-/HER2- or TNBC, as classified on routine pathology. We found no correlation between tumour size and HER3 expression (data not shown). Four patients consented to and underwent a second biopsy following initiation of neoadjuvant/systemic therapy.

**Table 1:** Patient characteristics

Pre-neoadjuvant therapy			
Subtype	ER + / HER2−	HER2 +	ER−/PR−/HER2−
n (%)	20 (58.8%)	11 (32.4%)	3 (8.8%)
Mean Age (years)	59.5	52.6	54
Range (years)	31–91	28–85	43–68
Tumour stage			
T1	0	1 (9%)	0
T2	14 (70%)	5 (45.5)	3 (100%)
T3	3 (15%)	4 (36.4%)	0
T4	3 (15%)	1 (9%)	0

Post-neoadjuvant therapy			
n	2	2	−
Mean Age (years)	58.5	47.5	−
Range	45–72	42–53	
Tumour stage			
T1	0	0	0
T2	1 (50%)	0	0
T3	1 (50%)	1 (50%)	0
T4	0	1(50	0

### Variable expression of HER3 in biopsies from breast cancer patients

**Fig. 2:**
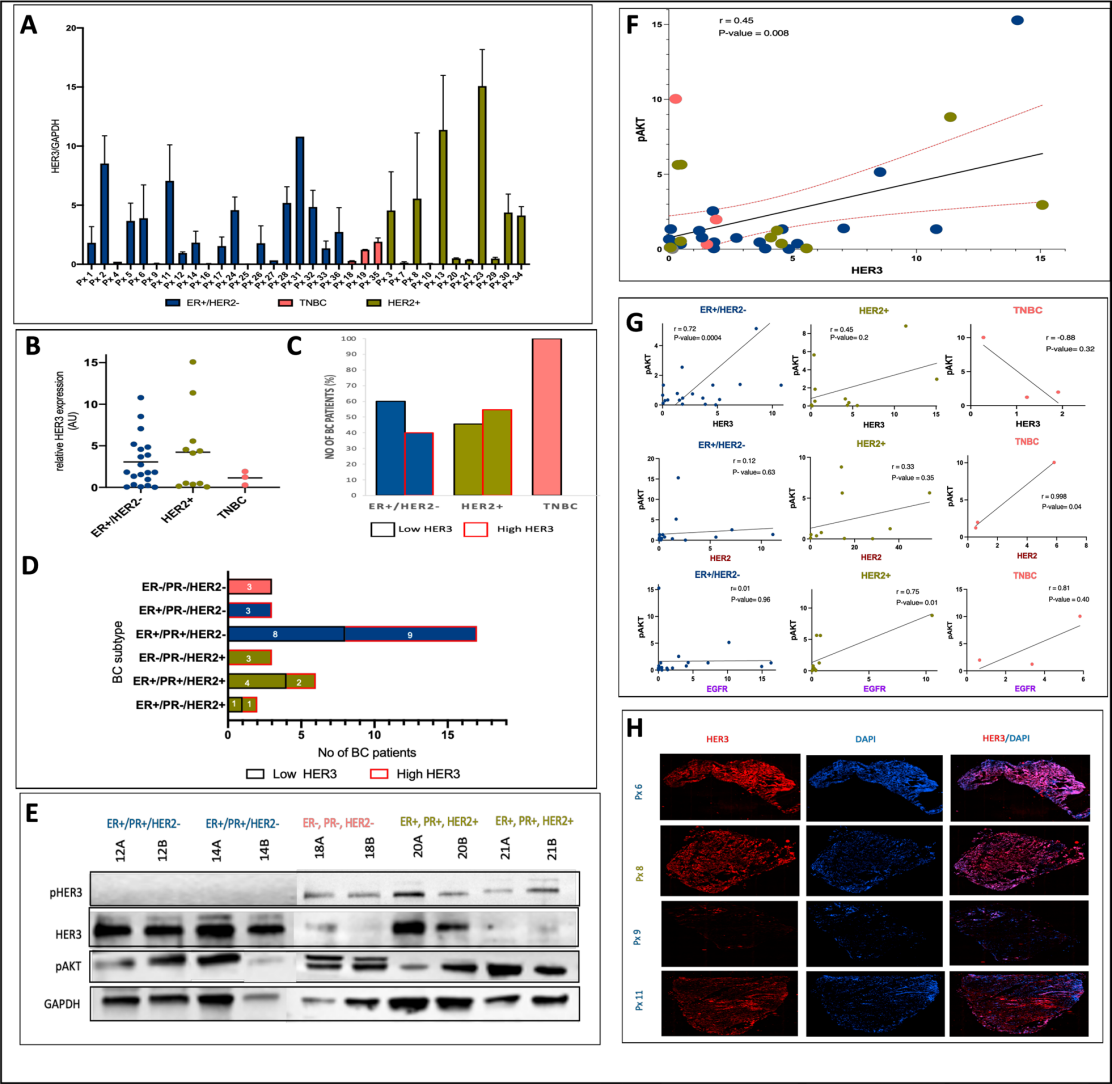
HER3 is variably expressed across different breast cancer subtypes at diagnosis. **A** Relative protein expression of HER3 in patients (n = 34) diagnosed with breast tumors and classified by hormone receptor and HER2 expression subtype with **B** mean HER3 expression based on receptor status and **C** patient frequency of low and high HER3 expressing tumors **D** Distribution of patients based on specific hormone status **E** Representative immunoblots of HER3 family receptors and downstream signaling demonstrating variable HER3 involvement in these breast cancer patients **F** Pearson correlation analysis showing a positive correlation between HER3 expression and phosphorylation of AKT in breast cancer patients **G** Pearson correlation between HER3 and phosphorylation of AKT as classified by intrinsic breast cancer subtype and **H** representative immunohistofluorescence analysis of HER3 expressing and non HER3 expressing breast cancer biopsies

We biochemically analyzed different subtypes of breast cancer to identify the extent of HER3 protein expression. We found large variability in HER3 expression across the three breast cancer subtypes (Fig. 2A). HER3 expression was highest in HER2+ breast cancer, with a mean of 4.2 ± 4.97 (AU), followed by the HR+ at 3.06 ± 3.02 (AU), and TNBC 1.14 ± 0.82 (AU). We found that 40% of the HR+ cancers had high HER3 expression, while 55% of HER2 + were classed as HER3 high tumors. The three TNBC tumors were all classed as low HER3 tumors (Fig. 2B). Further classification based on specific receptor status showed that the frequency of patients with low and high HER3 expressing tumors (defined as below or above mean HER3 expression) is evenly distributed in some but not all subtypes (Fig. 2C). However, the small sample size within certain subtypes may mask the extent of this variation. Representative immunoblots of HER3 and downstream signaling are depicted in Fig. 2D. As expected, we found that the PI3K/AKT pathway was predominantly activated in HER3+ breast cancer compared to HER3- breast cancer.

Next, we analyzed the correlation of HER3 expression with pAKT, a downstream mediator of cell survival, across all breast cancer subtypes and found a moderate correlation [r < 0.50] between them (Fig. 2E). Further correlation analysis based on breast cancer subtype (Fig. 2F) showed a strong correlation [r > 0.50] between HER3 and pAKT in ER+ breast cancer and a moderate correlation in HER2+ breast cancer. Interestingly, there was a strong negative correlation in TNBC. However, due to the small sample size, this effect may not be representative of the entire TNBC subtype. Of note is that one of the pathologically classed TNBC patients showed high HER2 expression as analyzed by western blot, thus possibly driving the relationship between HER2 and pAKT signaling. Correlation between HER2/EGFR expression and pAKT are shown in Supplementary Figure S1.

### HER3 is upregulated in response to neoadjuvant therapy in a subset of breast cancer patients

**Fig. 3:**
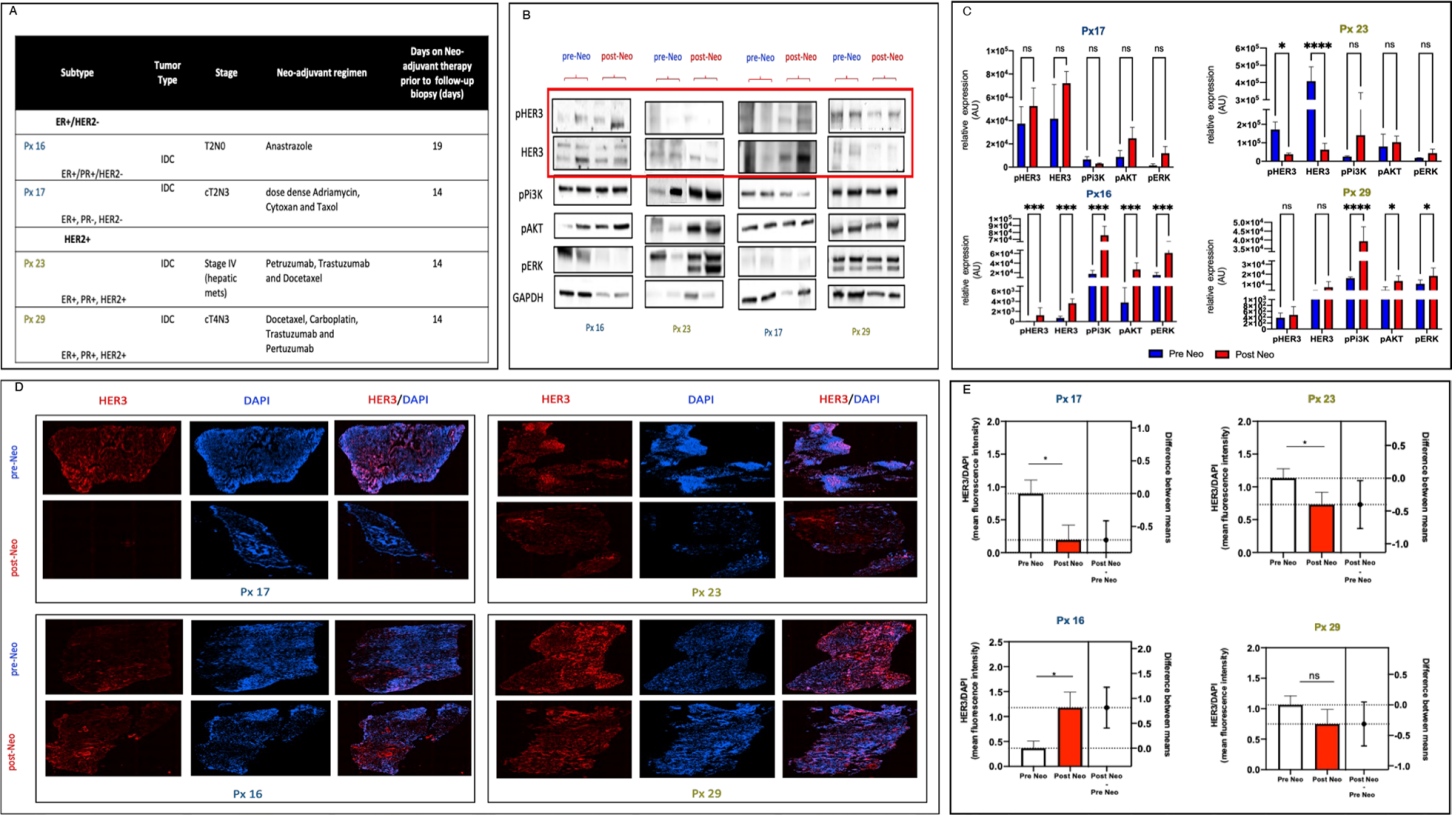
HER3 increases after neoadjuvant therapy in some but not all breast cancer tumors. **A** Patient characteristics and neoadjuvant regimens given **B** Representative immunoblots of HER3 and downstream signaling in patients pre and post neoadjuvant therapy and **C** Quantitative analysis of changes in these protein targets demonstrating variable response in the HER3 expression (n = 3). Data was analyzed using ANOVA analysis **D** Immunohistofluorescence of HER3 in patients receiving neoadjuvant therapy and **E** corresponding quantification analyzed using T-tests. *P < 0.05; **P < 0.01, ***P < 0.001, *****P < 0.0001

We next biochemically analyzed HER3 expression following initiation of systemic (neoadjuvant or palliative) therapy for the four study subjects who underwent a follow-up research biopsy. Systemic therapy was patient-specific and was selected by the patient’s oncology team per standard of care. The specific regimens are shown in Fig. 3A. The mean interval between initiation of systemic therapy and biopsy intervention was 15.25 ± 2.5 days.

Biochemical analysis of the biopsy samples pre and post neoadjuvant therapy revealed a varied response in HER3 expression as demonstrated in Fig. 3B. Interestingly, we saw significant HER3 increase (P = 0.0004) in Subject 16, who had ER+ (ER+/PR+ /HER2-) invasive ductal carcinoma. In contrast, Subject 23, who had HER2+ (ER+ /PR+ /HER2+) invasive lobular carcinoma, showed a significant HER3 decrease (P < 0.0001). The two other subjects, subject 17 with ER+ (ER+ /PR-/HER2-) invasive ductal carcinoma and Subject 29 with HER2 + (ER+/PR+/HER2+) invasive ductal carcinoma, showed no significant differences in HER3 expression from baseline.

Subject 16’s (ER+/PR+/HER2-) biopsy specimens, which showed significantly increased HER3 expression post systemic therapy initiation, also showed increased signaling in downstream mediators of the PI3K-AKT pathway (Fig. 3C), implicating HER3 as a potential active mechanism of continued cell survival in this cancer. While the sample size of patients analyzed post neoadjuvant therapy is very small, these findings may implicate HER3 as a feedback mechanism that is not related to a particular systemic therapy, but rather inherent to the tumor biology which may ultimately lead to treatment resistance.

We next analyzed HER3 expression by IHF in patients with both baseline and follow-up biopsy specimens, as shown in Fig. 3D. In corroboration with the immunoblot analysis, we found a significant increase in HER3 expression in Subject 16 (ER+) and a non-significant decrease in subject 29 (HER2+). Subject 23 (HER2+) showed a significant decrease in HER3 expression (P < 0.0001), while Subject 17 (ER+) showed a significant decrease in HER3 expression (P < 0.032). The slight discrepancy between the western blot and IHF in this subject may be related to the specific site of the tumor that underwent sampling, and potentially indicates a heterogeneous response within the tumor.

Given the notorious dynamic response of HER3, we investigated if HER3 increase is controlled transcriptionally in response to different neoadjuvant regimens. To this end, we performed q-PCR HER3 analysis (data not shown) and found modest changes at the transcript level of HER3 expression in pre- and post-neoadjuvant treated patients.

Representative diagnostic images prior to treatment and following initiation of neoadjuvant therapy (performed at the time of the follow-up research biopsy) are shown in Supplementary Figure S2. MRI and mammogram diagnostic images of patients 16, 17 and 29 can be found in Supplementary Figure S3. There are no ultrasound images available for subject 23.

### Analyzing HER3 changes to neoadjuvant therapy to understand the potential role of HER3 PET imaging in response prediction

It has previously been shown that breast cancers with a complete pathologic response after NAT have a lower recurrence rate compared to those with a partial response [35]. One potential mechanism of tumor recurrence and/or metastasis may involve HER3-mediated cell growth and survival pathways among residual tumor cells. Molecular characterization of the tumor response to NAT poses an ideal window of opportunity for identifying patients who may develop HER3 mediated resistance. However, to date, molecular characterization of response to NAT is not routine practice nor completely understood.

To further validate our findings and gain more insight into the biological mechanisms, we analyzed the GSE122630 dataset from patients with longitudinal data from primary breast cancer biopsies at four time-points during neoadjuvant therapy. The cohort consisted of 41% (14/34) histological Grade 2 breast cancer and 59% (20/34) Grade 3. 76% (26/34) were HER2- and 23.5% (8/34) were HER2+ . Patients received fluorouracil, epirubicin and cyclophosphamide (FEC) and docetaxel with Herceptin when appropriate. Three patients received paclitaxel, one patient received additional carboplatin, one patient received Epi-cyclophosphamide and paclitaxel, and one patient received docetaxel and cyclophosphamide [23].

**Fig. 4:**
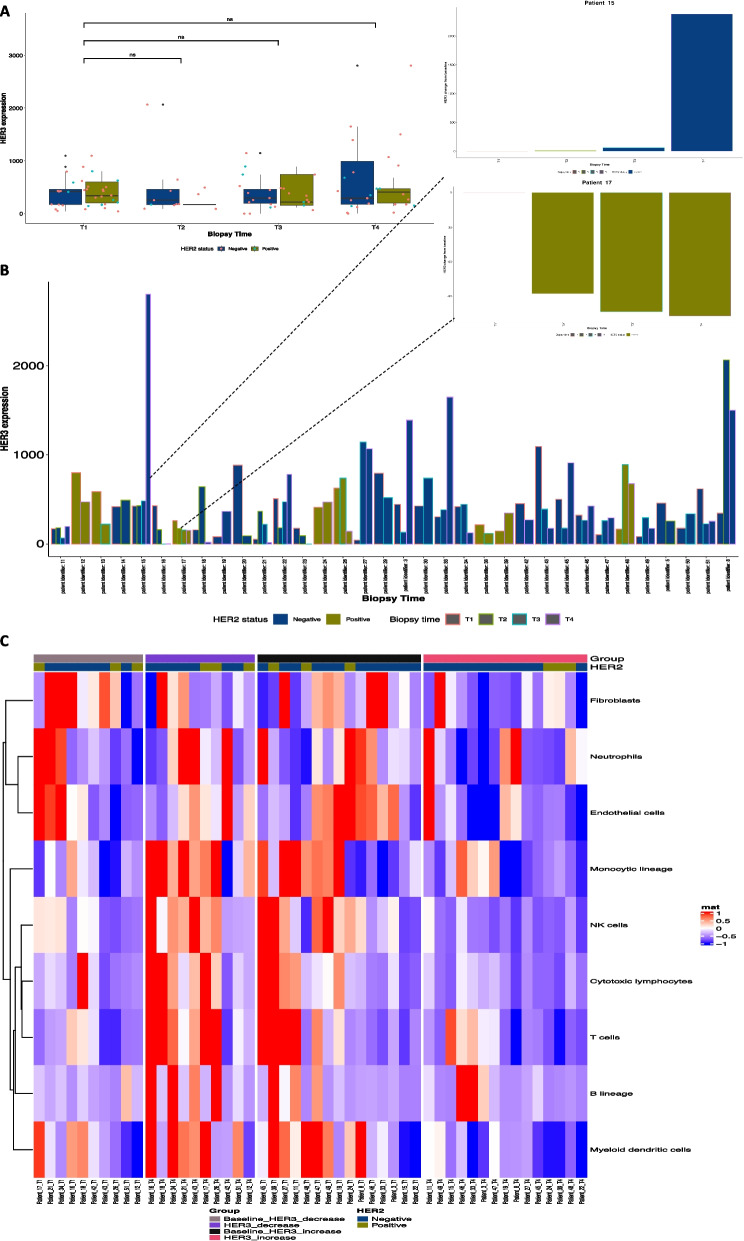
Assessment of HER3 in patients from the GSE122630 study pre and post neoadjuvant therapy. **A** HER3 expression at baseline (T1), at two weeks during neoadjuvant therapy (T2), mid-neoadjuvant therapy (T3) and at surgical resection (T4) **B** HER3 expression per patient at all timepoints where a biopsy was available with insets showing an example of a patient with HER3 decrease and a patient with HER3 increase **C** Microenvironmental cell populations in patients with HER3 decrease and patients with HER3 increase at T4 (surgical resection)

Bulk transcriptomic analysis of HER3 at the time-points examined revealed no significant difference in HER3 expression at transcriptomic level (Fig. 4A). However, on a per sample basis and similar to our cohort, HER3 expression varied across the patient population with variable responses to NAT (Fig. 4B). Per patient HER3 change from baseline analysis are shown in Supplementary Figure S4. Due to the dynamic nature of HER3 expression, and the variable responses seen longitudinally, only patients that had a biopsy at baseline and at surgical resection (T4) were further analysed. Of the 25 patients that had a T1 and T4 biopsy, 40% (10/25) showed a decrease in HER3 expression and 60% (15/25) showed an increase in HER3 expression. Of the patients that showed an increase in HER3 expression, 80% were HER2- and 20% were HER2+. Of the patients that showed a decrease in HER3 expression, 70% (7/10) were HER2- and 30% were HER2+.

We next sought to gain an understanding of the molecular mechanisms governing the differential responses in these patients. Although chemotherapy has been traditionally thought of as immunosuppressive, current research has challenged this dogma [36]. Studies have shown that chemotherapeutic treatment can also activate the immune system by inducing immunogenic cell death in tumours and promoting damage- associated molecular pattern (DAMP) molecules. Furthermore, HER2-HER3 can cause potent downstream signaling through the PI3K-AKT pathway which been shown to play a significant role in innate and adaptive immune responses as well as in cancer tumorigenesis [37]. To this end we performed Microenvironmental cell population analysis (MCP) which measures 8 immune populations and 2 stromal cells [25]. Interestingly, we found an immunostimulatory response in patients that show no HER3 upregulation post NAT. Conversely, there was a decrease in the expression of innate and immune cells including monocytes, NK-cells, cytotoxic lymphocytes and B-cells, in patients with a HER3 increase post NAT.

Thus, HER3 expression shows variable expression across breast cancer subtypes with patient specific dynamic longitudinal responses to NAT. Dichotomization based on HER3 increase or decrease post NAT shows differential microenvironmental responses highlighting a potent involvement of immune regulation.

### Upregulation of HER3 following neoadjuvant therapy causes immunosuppression

**Fig. 5:**
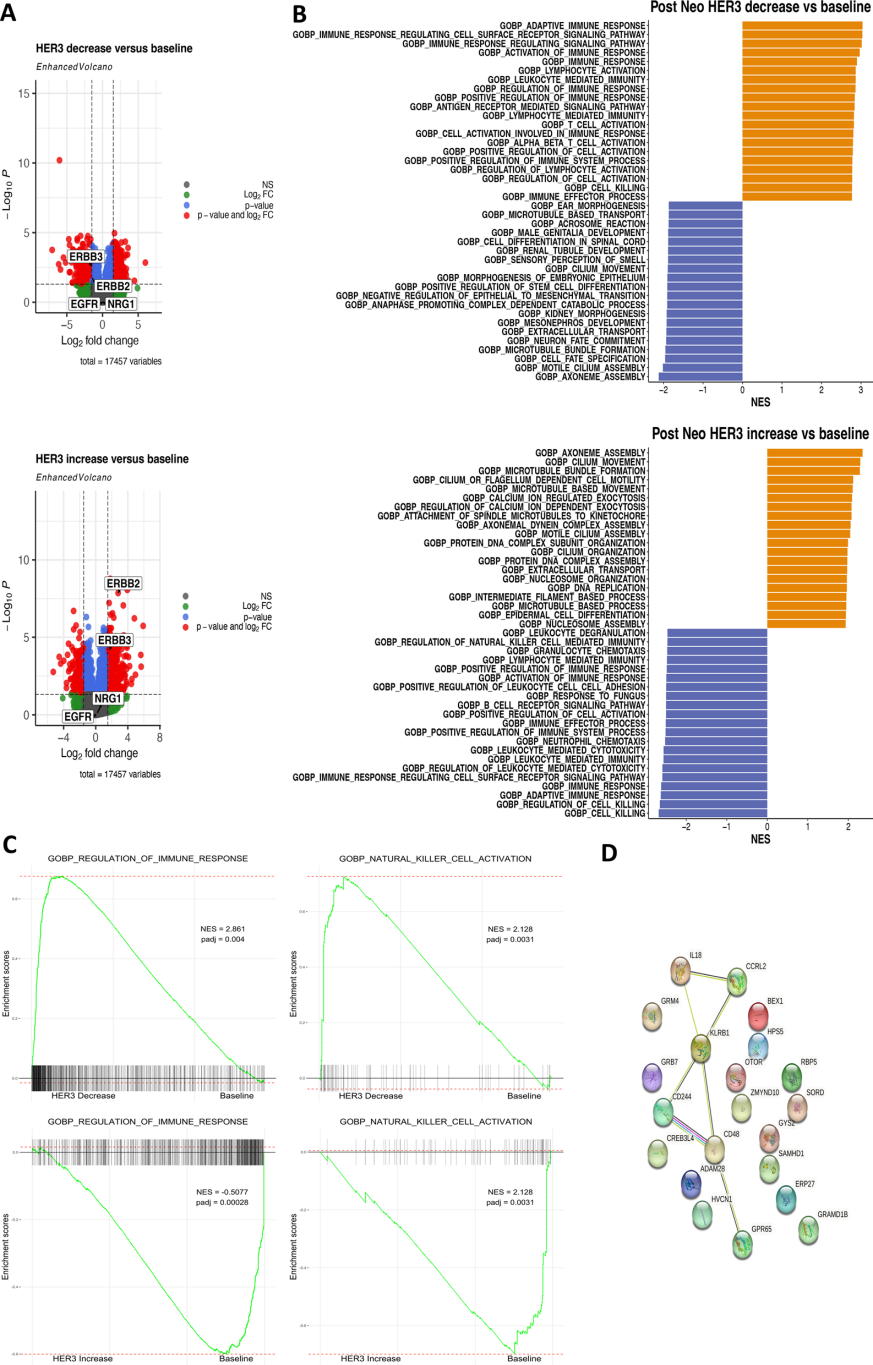
Differential biologies in patients with HER3 increase post neoadjuvant therapy. **A** Differential gene expression in patients with HER3 decrease or HER3 increase at T4 (surgical resection). **B** Top 20 most upregulated and downregulated genesets in patients that showed a HER3 increase or decrease post neoadjuvant therapy. **C** Enrichment plots showing upregulation and downregulation of immune response in patients with HER3 decrease or HER3 increase post neoadjuvant therapy. **D** String network formed by the top 11 most upregulated genes in patients that showed a HER3 decrease post-neoadjuvant therapy

Having demonstrated a differential tumour microenvironment post NAT, we set to further explore the biological mechanisms governing these changes. We next performed differential gene expression (DGE) in patients with and without HER3 upregulation. DGE analysis between T1 and T4 in patients that showed an increase in HER3 expression revealed a significant increase in HER2 and HER3 but not in EGFR or NRG1. Contrary, HER3 and HER2 were significantly decreased in patients that showed a HER3 decrease at T4 (Fig. 5A). GSEA on the differentially expressed genes was performed using the biological processes collection from the msigdbr-database. Figure 5B shows the corresponding top 20 most up-regulated and down-regulated gene sets in patients with and without HER3 increase post NAT. Interestingly, in patients who did not show HER3 upregulation post NAT, all of the 20 most significantly enriched genes are involved in activation of immune pathways. Of the most downregulated pathways, patients with HER3 decrease show a negative enrichment in epithelial to mesenchymal transition, positive regulation of stem cell differentiation, possibly inferring cellular processes not involved in tumor progression. In contrast, all the top 20 most downregulated pathways, in patients that showed a HER3 increase post NAT, show a decrease in regulation and activation of innate and adaptive immune signalling (Fig. 5B). Conversely, upregulated pathways include cell division processes such as nucleosome organisation, DNA replication and a number of microtubule-based processes. Microtubules serve as intracellular cytoskeleton and are involved in cellular activities such as cell division, growth and movement. In cancer, microtubules have been associated with cancer metastasis whereby the reorganization of microtubules allows for morphological changes associated with cell movement, contributing to cancer migration, invasion, and epithelial-to-mesenchymal transition [38].

Enrichment analyses further confirmed a significant upregulation of immune responses and Natural killer cell (NK-cell) activation in patients who do not upregulate HER3 post NAT, while the reverse was seen in patients with HER3 upregulation (Fig. 5C).

Lastly, we used the STRING database (string-db.org) to identify and visualise interactions of up- regulated genes and found that a network was formed around NK-cell activation and modulation in patients that showed a HER3 decrease post neoadjuvant therapy (Fig. 5D).

### HER3 increase post neoadjuvant therapy may increase tumorigenic potential

**Fig. 6:**
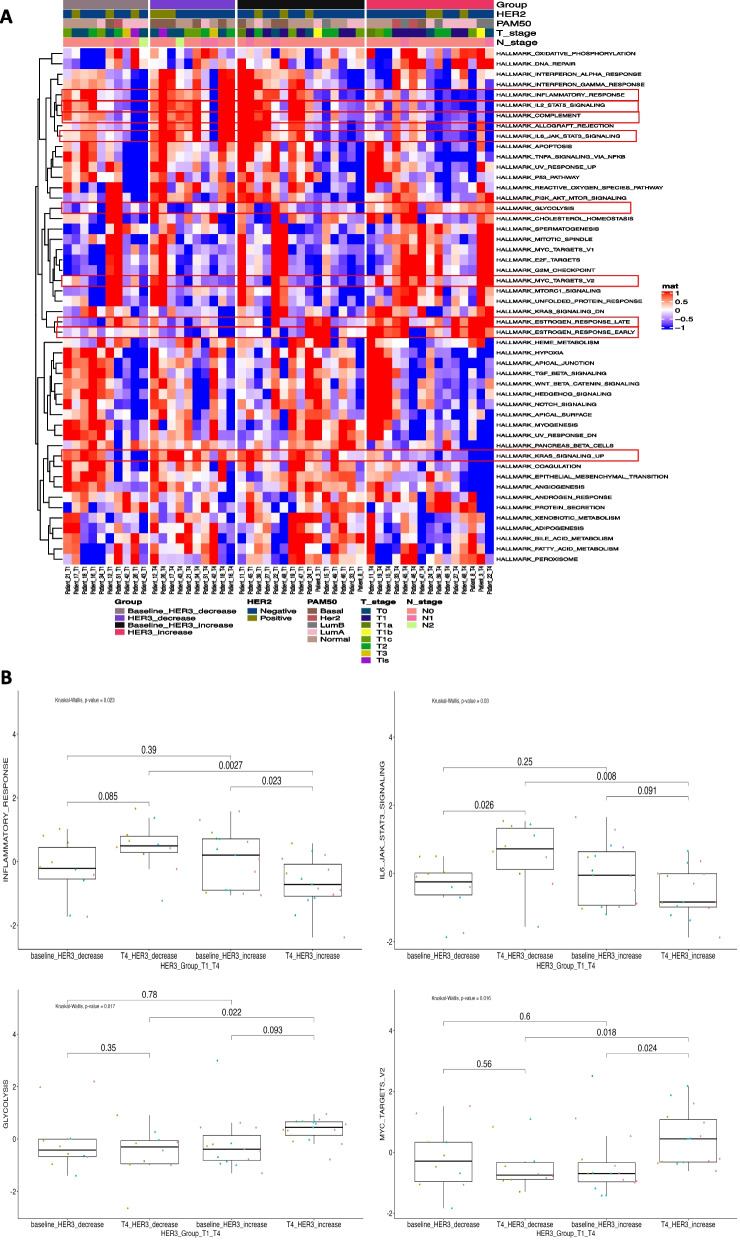
**A** Heatmap of the hallmark gene sets between patients with decreased or increased HER3 expression at T1 and T4 (n = 25) with red inset depicting the statistically significant hallmarks and **B** Boxplots showing comparison between patients with an increase or a decrease in HER3 post NAT in a select number of significantly differential pathways from the Hallmarks gene sets as analyzed using Kruskal–Wallis test. *T*-test between groups were carried out and *P*-values < 0.05 were considered statistically significant

We next performed single sample GSEA (ssGSEA) analysis using the hallmark gene sets [39] and confirmed a differential biology in patients that showed increased HER3 expression post NAT compared to those that did not at T4 (Fig. 6A). Out of the 50 hallmarks, we found no significant difference at baseline between patients that showed a HER3 increase versus HER3 decrease post NAT, regardless of HER2 status. While this finding is not necessarily surprising it does highlight the difficulty in the identification and stratification of patients at diagnosis. In agreement with previous results shown within we found differential responses after NAT in patients with or without HER3 increase. Specifically, patients with HER3 upregulation post NAT had a decrease in immune signaling and increases in proliferation, metabolic, and signaling pathways as seen in the associated boxplots in Fig. 6B.

Interestingly, we found that in patients with HER3 decrease post NAT, there is a decrease in estrogen response early and estrogen response late gene sets. These gene sets consist of 200 genes that react early or late, respectively, to estrogen. Studies have demonstrated that a high estrogen response early score is associated with better prognosis in BC [40, 41]. In contrast to our results, it was found that in ER+ breast cancer a low estrogen response score tumor was significantly infiltrated with both anti- and pro-cancer immune cells, as well as B cells, including CD8^+^ T cells, CD4^+^ T cells, M1 macrophages, dendritic cells, regulatory T cells, Th2 cells, M2 macrophages, and plasma cells, and associated with enhanced cytolytic activity score [41]. Thus, the relationship in respect to HER3 upregulation needs to be explored further to determine the association with therapy response.

Taken together our results support the hypothesis that patients in which HER3 upregulation occurs post NAT have a suppressed immune response. While the upregulation of HER3 may be an early predictor of tumor resistance as evidenced by the increase in cellular division in these patients, future studies are needed to determine the complex mechanisms governing these different responses.

## Discussion

Currently, one of the primary goals of NAT is to decrease the tumor size and burden of locally advanced breast cancer in order to minimize the surgical resection and to increase the likelihood of breast conservation therapy. Biomarkers that can predict and guide targeted neoadjuvant therapy regimens would greatly facilitate the goals of minimizing and overcoming treatment resistance.

HER3, although not commonly mutated or overexpressed, can form potent heterodimers with all its family members to provide cell survival and proliferation advantages, contributing to treatment failure.

We show that HER3 expression is variable in breast cancer samples at diagnosis. Furthermore, we show a variable response to therapy, and that up- or down-regulation is not subtype specific, as previously demonstrated [42, 43]. Our study suggests that HER3 expression and up-regulation following initiation of NAT occurs in ER+ as well as HER2+ breast cancer. Although, no TNBC patients were analysed post NAT in this study, results from our group and others have shown the active involvement of the HER3/EGFR signalling axis in TNBC resistance to different targeted therapeutics. We found that HER3 up-regulation can occur with distinct therapeutic regiments, implicating HER3 as one of the universal feedback mechanisms possibly involved in cancer resistance. Thus, the results presented within are in agreement with published literature demonstrating HER3 involvement in breast cancer therapy resistance in ER+ , HER2+ and TNBC subtypes [44].

Bioinformatic approaches in patients with and without HER3 upregulation post NAT revealed differential underlying biologies. Specifically, HER3 upregulation post NAT was associated with immunosuppression as evidenced by decreased immune cell populations such as T cells, NK-cells, B-cells and monocytes. In contrast, immune activation and stimulation was seen in patients who did not show HER3 upregulation post NAT. Previous studies have demonstrated the dynamic effects of NAT in breast cancer patients with infiltrating tumor lymphocytes, CD8 + cells and upregulation of immune stimulatory signatures associated with pathological complete response [45–47].

The molecular profile after as early as two weeks of neoadjuvant/systemic therapy has been suggested to be predictive of treatment response, but not pre-therapy [48]. Our study suggests that neoadjuvant/systemic therapy causes a rapid patient-specific molecular adaptation. Thus, although baseline molecular profile may provide an initial therapeutic avenue, post assessment may provide early therapeutic tailoring. Changes at protein level make imaging technologies ideal for assessing tumor response and pave the road to HER3-PET imaging in the clinical setting, through non-invasive, repeat PET imaging of the HER3 receptor across the full burden of disease. Although, HER3 cannot be definitively implicated in resistance to systemic therapy, it opens a novel paradigm that may be used early in the assessment of tumor response and forms a novel avenue for tumor targeting. Further analyses need to explore the complex underlying mechanisms leading to immune down-regulation in these patients.

**Fig. 7:**
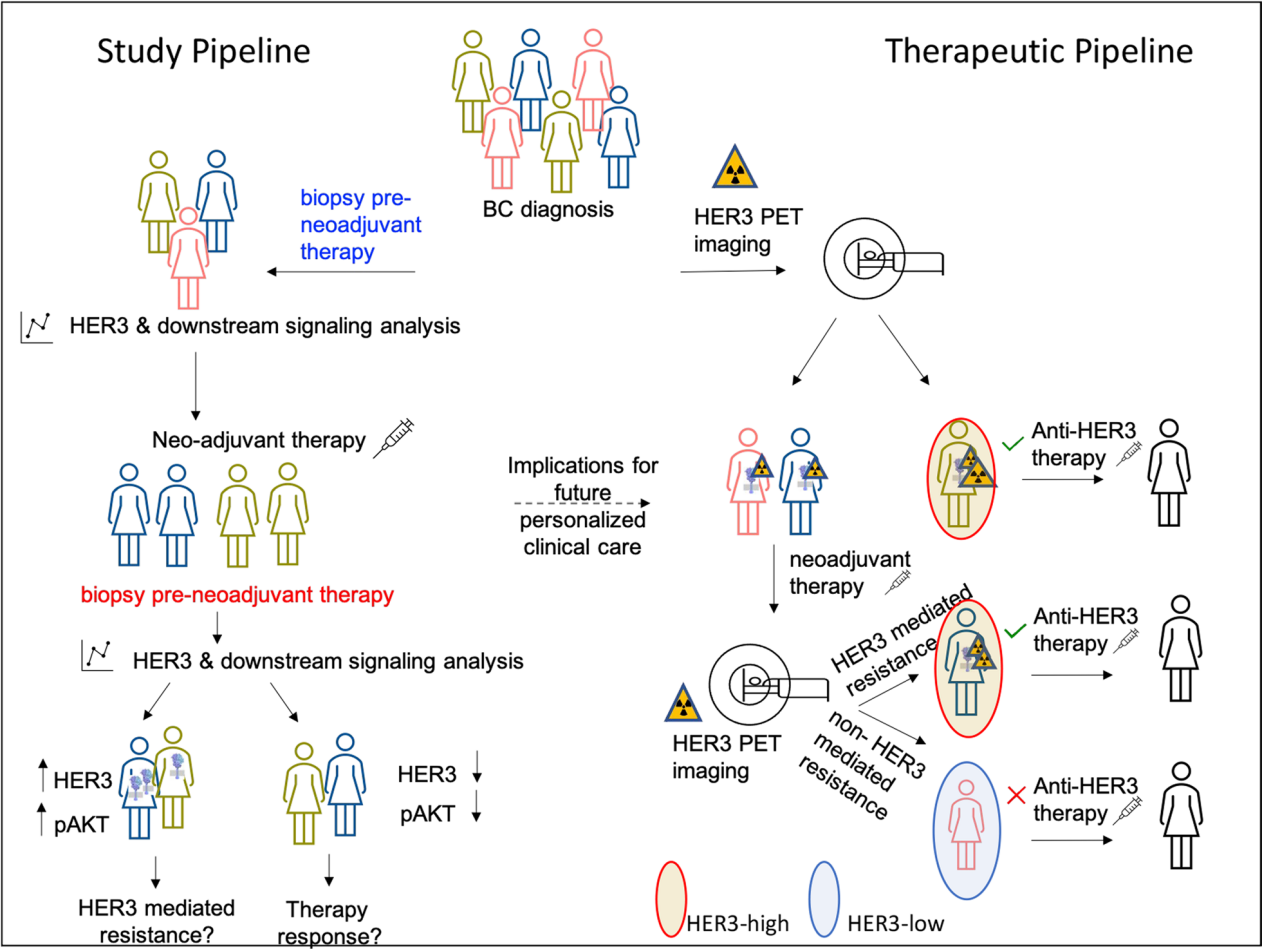
Schematic of study pipeline (left) and proposed diagnostic and therapeutic approaches for breast cancer (right)

In conclusion, our study shows that HER3 is variably expressed in breast cancer, and that its up-regulation can be detected after NAT, potentially offering an early indication of treatment failure. Long term follow-up and larger clinical studies are needed to further implicate HER3 as a mechanism of resistance to neoadjuvant therapy, although mounting evidence exists in the adjuvant setting. Tumor assessment should include HER3 characterization pre and post therapy initiation, and novel imaging modalities may allow for non-invasive detection for a personalized therapeutic targeted approach as summarised in the schematic in Fig. 7.

### Supplementary Information

**Additional file 1**.

## Data Availability

The datasets generated during the current study are available from the corresponding author on reasonable request. The datasets analysed during the current study are available in the GEO Omnibus (GSE122630) and cBioPortal repositories.

## Abbreviations

AKT: Protein Kinase B
CRAN: Comprehensive R Archive Network
EGFR: Epidermal Growth Factor Receptor
ER: Estrogen Receptor
ER −: Estrogen Receptor Negative
ER +: Estrogen Receptor Positive
GSE: Gene Expression Omnibus Series
GEO: Gene Expression Omnibus
HER2: Human Epidermal Growth Factor Receptor 2
HER2 −: Human Epidermal Growth Factor Receptor 2 Negative
HER2 +: Human Epidermal Growth Factor Receptor 2 Positive
HER3: Human Epidermal Growth Factor Receptor 3
MAPK: Mitogen-Activated Protein Kinase
MRI: Magnetic Resonance Imaging
NCBI: National Center for Biotechnology Information
PCR: Polymerase Chain Reaction
PET: Positron Emission Tomography
PI3K/AKT: Phosphoinositide 3-Kinase/Protein Kinase B
PAM50: Prediction Analysis of Microarray 50
PR: Progesterone Receptor
PR −: Progesterone Receptor Negative
PR +: Progesterone Receptor Positive
RAS: Rat Sarcoma
RIPA: Radioimmunoprecipitation Assay
RNA: Ribonucleic Acid
ssGSEA: Single-Sample Gene Set Enrichment Analysis
TNBC: Triple-Negative Breast Cancer
TNF: Tumor Necrosis Factor
