# Supplementary material for: A prospective study of HER3 expression pre and post neoadjuvant therapy of different breast cancer subtypes: implications for HER3 imaging therapy guidance
Source: Breast Cancer Res. 2024 Jun 29;26:107. doi: 10.1186/s13058-024-01859-w (PMC11218108; doi:10.1186/s13058-024-01859-w)
Supplement: Supplementary file 1 — Additional file 1. [file 13058_2024_1859_MOESM1_ESM.docx]

Supplementary


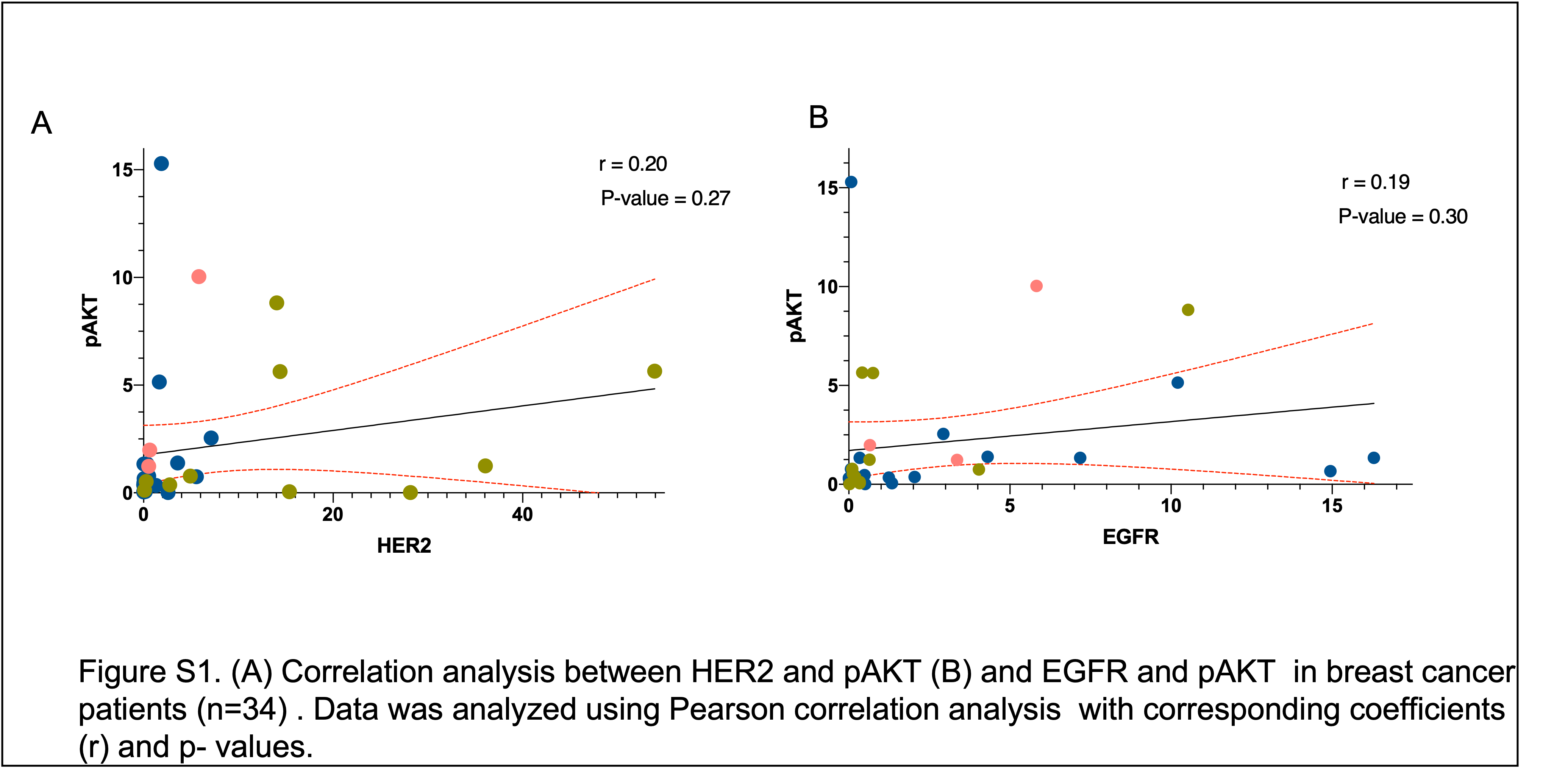


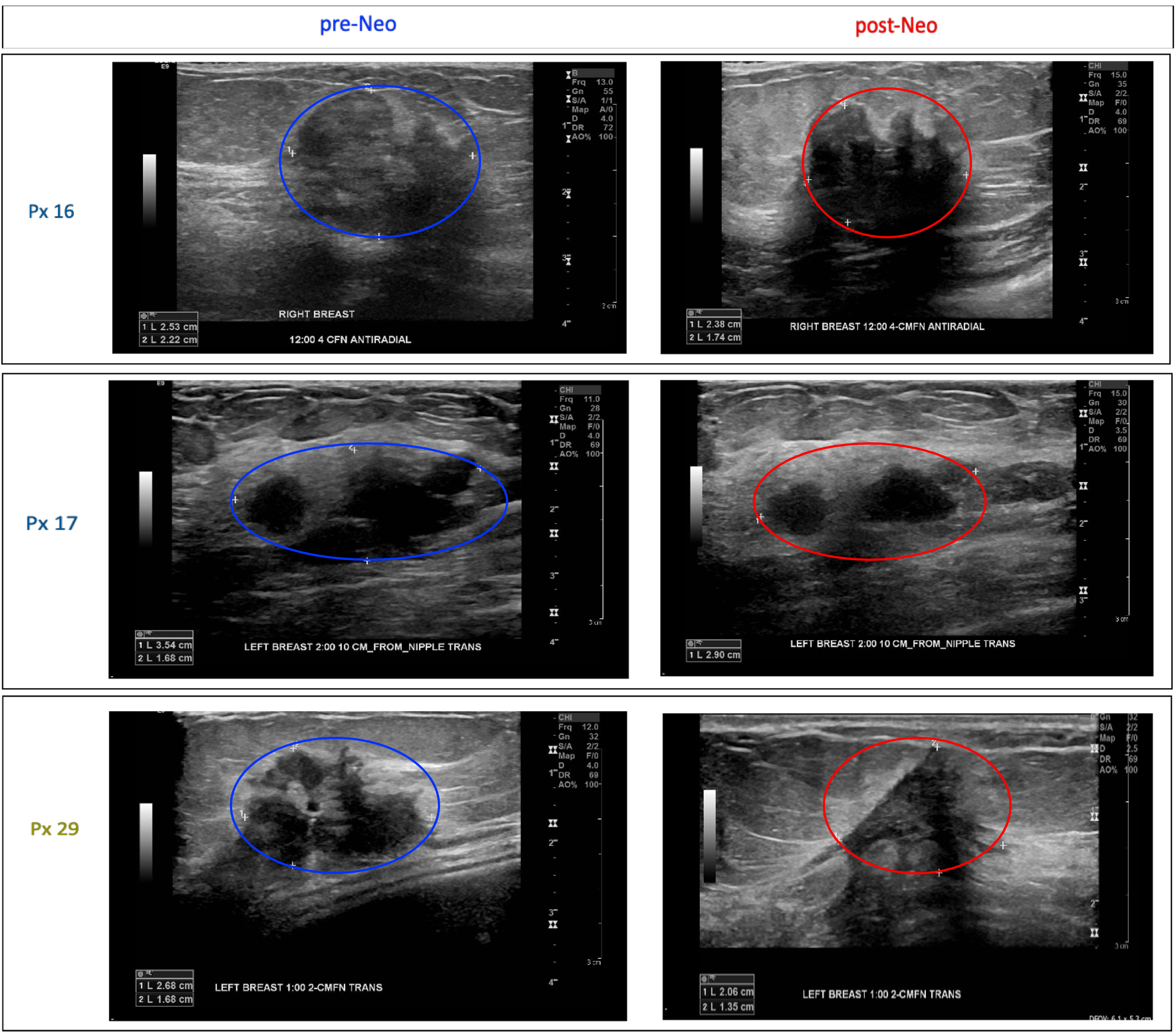


Figure S2. Ultrasound images of pre and post therapy in breast cancer patients

Figure S3. Representative clinical images pre and post neoadjuvant therapy for a breast cancer patient (Px 29) who received Docetaxel, Carboplatin, Trastuzumab and Pertuzumab. Diagnostic MRI performed pre (top) and following (bottom) initiation of neoadjuvant treatment.
